# Biologically inspired optimization of construction sector eco industrial park networks using food web metrics

**DOI:** 10.1038/s41598-026-54667-x

**Published:** 2026-05-24

**Authors:** Olcay Genc, Atıl Kurt

**Affiliations:** 1 Department of Civil Engineering, Faculty of Engineering, Bursa Uludag University, Nilufer, Bursa, Turkey; 2 Department of Industrial Engineering, Alanya Aladdin Keykubat University, Alanya, Antalya, Turkey

**Keywords:** Bio-inspired design, Circular economy, Construction sector, Ecological network analysis, Eco-industrial parks (EIP), Food webs, Industrial symbiosis, Industrial ecology, Optimization, Ecology, Ecology, Mathematics and computing

## Abstract

Industrial symbiosis (IS) and eco-industrial parks (EIPs) are increasingly promoted as practical pathways to circular economy transitions in resource-intensive sectors such as construction, where diverse waste streams, quality variability, and fragmented supply chains often constrain the number and stability of feasible exchanges. Building on biomimetic design principles, this study investigates whether construction-sector EIP networks can be optimized to better resemble selected structural patterns observed in biological food webs, and how optimization choices and participation rules influence the resulting network topology. Using a construction symbiosis database and five theoretical exchange scenarios, scenario-constrained optimization models are formulated to seek proximity to detritus-inclusive biological food-web reference values. Four objective function types (OFTs), representing alternative ways of aggregating multi-metric deviation from benchmarks, were tested in two parallel model families: one excluding connectance from the objective set and one explicitly targeting connectance to assess its conditioning role. A genetic algorithm was used to optimize the scenario-constrained network models and efficiently explore the large combinatorial solution space. Results show that structural proximity to the selected food-web benchmarks is configuration-dependent. Scenario rules and OFT choice systematically steer solutions toward distinct network morphologies, producing clear trade-offs across metrics rather than uniform improvement. Across best-performing configurations, the ratio of waste-providing to waste-receiving firms was comparatively close to benchmark levels in some cases but showed notable deviations in others, while link density and cyclicity exhibited persistent deficits, indicating that achieving dense, highly cycling structures is challenging under construction-specific feasibility constraints. Explicitly including connectance reduced the tendency of some OFTs to converge to extreme connectivity regimes and yielded more balanced metric profiles, highlighting connectance as a structuring constraint that limits extreme connectivity rather than as evidence of ecological realism. Reciprocity-oriented participation rules, particularly those requiring receiver firms to also provide exchanges, were associated with more benchmark-consistent solutions under certain OFT and connectance-included combinations, rather than uniformly dominating across all cases. For practice, the findings suggest that structurally informed bio-inspired EIP planning may benefit from treating connectance as a controlled design parameter and considering reciprocal participation policies where they are compatible with the selected objective formulation and feasibility constraints. Future research should integrate exchange quantities, cost and quality constraints, and uncertainty dynamics, and should report Pareto-efficient solution sets to support stakeholder selection and implementation.

**Supplementary Information:**

The online version contains supplementary material available at 10.1038/s41598-026-54667-x.

## Introduction

Industrial production and consumption systems remain major drivers of environmental pressure, not only through direct emissions but also through persistent material losses and accumulating waste streams. In response, industrial ecology has long argued that industrial systems should be understood and redesigned as metabolic systems, with sustainability depending on the circulation of materials and energy rather than on linear throughput^1^. This framing has motivated practical strategies that aim to retain value in materials through reuse, recovery, and reconfiguration of inter-firm resource flows, especially within geographically bounded industrial zones^2^. Within this tradition, eco-industrial parks (EIPs) have been proposed as an actionable organizational form for accelerating industrial circularity. EIPs seek to reduce net resource demand and environmental impact by enabling firms to share resources, services, and by-products, thereby generating system-level efficiencies that are difficult to achieve through isolated firm-level interventions^3^. The core mechanism that operationalizes this ambition is industrial symbiosis (IS), commonly understood as physically grounded exchanges of materials, energy, water, and by-products among co-located or regionally connected firms^4^. Research has progressively clarified that the formation of such exchanges depends not only on technical feasibility but also on institutional conditions, information infrastructures, and actor expectations^5,6^.

Early EIP initiatives were shaped both by the conceptual appeal of ecosystem analogies and by the practical challenge of translating that analogy into replicable design and governance approaches. In a seminal synthesis, Chertow (2000) organized early EIP and IS efforts into a taxonomy of exchange types and highlighted tools such as input–output matching and materials budgeting as supports for implementation^4^. Chertow’s historical analysis of industrial symbiosis stressed the importance of recognizing and nurturing “kernels” of symbiosis that already exist, rather than assuming that planned projects will self-assemble into high-performing networks (Chertow^7^. A parallel stream of work has emphasized that EIPs are best interpreted as networks rather than as collections of independent pairwise transactions. This network perspective aligns with broader findings from social and complex network research suggesting that system-level outcomes depend on global topology, not solely on local links^8–10^. In EIP research, this insight has encouraged analytical approaches that treat inter-firm exchanges as weighted or directed graphs and use network measures to assess robustness, vulnerability, and sustainability over time.

Importantly, the network turn in EIP scholarship has not been purely methodological. It is also conceptual, rooted in the longstanding industrial ecology analogy that industrial systems can be interpreted as industrial ecosystems^11,12^. From this viewpoint, the structure of inter-organizational exchanges matters because ecological systems derive resilience and adaptive capacity from a combination of diversity, redundancy, and cycling pathways. This ecological logic has been made explicit through food web analysis (FWA) and related ecological network concepts, which offer formal metrics for describing directed interaction networks^13,14^. For example, FWA has been used to interpret the industrial ecosystem analogy as a measurable set of structural properties^13–15^, with potential implications for long-term network sustainability—the ability of an EIP configuration to preserve environmental, economic, and social benefits amid shocks and actor turnover^16,17^.

The biomimetic orientation of this literature is reinforced by a broader design discourse in which nature is framed as a source of functional principles for sustainability and innovation^18,19^. Methodological proposals in biomimicry and eco-design further stress that nature-inspired design requires careful translation from biological systems to engineered systems, including explicit criteria for what is being mimicked and how success is evaluated^20^. In EIP contexts, such translation motivates not only descriptive comparisons to ecological systems but also computational frameworks that embed ecological metrics within optimization. Optimization has therefore become an increasingly prominent instrument for EIP design and redesign, as it can systematically explore large combinatorial spaces of candidate exchanges while honoring operational constraints and stakeholder preferences. The diversity of objectives in EIP problems is well documented, including economic criteria, emission reduction, and energy exchange performance, as well as stakeholder-oriented value creation challenges^21,22^. As a result, EIP optimization research has expanded into multi-criteria formulations and design-support methods, supported by reviews that synthesize optimization approaches used specifically for eco-industrial park design^23^. Complementary methodological work has proposed frameworks for EIP design and optimization that integrate network structure with engineering feasibility and sustainability considerations^24^. Within this optimization strand, a particularly distinctive contribution has been the explicit integration of ecological food web metrics into industrial network design. Studies have used ecological structural indicators, such as connectance, linkage density, and trophic-role-based descriptors, to generate industrial networks that are not only feasible in exchange terms but also “ecologically plausible” in structure^25,14,15^. This direction is supported by ecological theory emphasizing that food web structure can be meaningfully summarized through network metrics and that connectance and size jointly influence emergent properties^26^. Related ecological modeling work also emphasizes the relevance of cyclic pathways in ecological food webs, which motivates attention to closed-loop pathways in industrial exchange systems (Fath and Halnes,^27^. At the same time, the literature cautions against assuming that any single ecological metric can serve as a sufficient proxy for ecological realism or long-term network performance. In biomimetic EIP design, for instance, empirical work shows that maximizing a single structural descriptor such as connectance does not necessarily yield an ecologically ‘better’ or more stable configuration^28^, consistent with broader food web theory demonstrating that stability depends not only on linkage density but also on interaction types and strength distributions^29^; Angelis^30^;^26,31,32^. In parallel, socio-ecological and practice-oriented research on transitions from conventional industrial zones to eco-parks emphasizes that many zones fail to meet eco-park expectations when symbiotic linkages are absent or limited, and that implementing theoretically designed eco-parks remains risky because participant objectives, institutional capacity, and operational uncertainty shape whether proposed exchanges can persist in practice Chertow^5,7,17,24,33,34^.

Although the literature establishes robust foundations for the taxonomy and institutional dynamics of industrial symbiosis^6^; Chertow^7,35^, network and ecology informed approaches to EIP evaluation^25,13–15,36^, and optimization-driven EIP design^23,24^ there remains a specific methodological gap at the intersection of biomimetic evaluation and optimization: few studies explicitly optimize industrial exchange configurations to achieve simultaneous proximity to a multi-dimensional biological benchmark vector of food web metrics. Put differently, prior work has demonstrated how ecological metrics can describe or benchmark industrial exchange networks^25,13–15^, how multi-criteria EIP design methods can address stakeholder and sustainability concerns^21,22,17^, and how connectance and cyclicity-focused or ecology-inspired objectives can be embedded within optimization^28,37,24^. However, a systematic optimization formulation that evaluates industrial exchange configurations through the simultaneous minimization of deviations across a suite of ecological network benchmarks, rather than maximizing a single structural indicator, remains underdeveloped. This study casts eco-industrial park design as a scenario-constrained network optimization problem aimed at selecting a feasible portfolio of inter-firm exchanges such that the induced industrial exchange network exhibits minimum multivariate deviation from selected biological food-web reference values, thereby approximating selected structural properties observed in food-web networks. By reframing biomimetic EIP design as a multi-metric structural benchmark-proximity problem, the study provides a more explicit operational basis for comparing industrial symbiosis configurations against selected ecological network metrics, while remaining grounded in verifiable biological reference values and established EIP design constraints.

The remainder of the paper is organized as follows: Sect.  2 presents the methodology, incorporating both the materials and scenario setup as well as a detailed description of the research approach and analytical procedures. Section  3 presents the results and Sect.  4 offers the discussion of the EIP scenarios, including comparisons with biological food webs. Lastly, Sect.  5 concludes the paper.

## Methodology

The study demonstrates the effects of nature-inspired objectives and constraints on the optimization of industrial networks, based on five theoretical eco-industrial park scenarios associated with the construction industry.

The methodology of this study is structured to examine the design and evaluation of construction-sector eco-industrial park (EIP) scenarios through a bio-inspired network perspective. EIP scenarios describes the eco-industrial park (EIP) scenarios developed for the construction sector, including the underlying exchange rules and scenario logic. Ecological network analysis (ENA) introduces the ecological network analysis framework adopted in the study, including the food web representation and the set of food web metrics used to benchmark industrial symbiosis networks against natural ecosystems. Mathematical modelling presents the scenario-constrained optimization approach, detailing the mathematical formulation used to configure EIP networks toward ecological benchmark values, together with the underlying assumptions. Genetic algorithm (GA) describes the solution methodology based on a genetic algorithm, outlining the chromosome structure, fitness evaluation, and search procedure employed to identify near-optimal network configurations.

### EIP scenarios

The construction industry represents a major contributor to the global economy^38^ and is also among the sectors generating significant waste during material production. However, IS research in the construction context has largely focused on demolition waste and the conventional reuse of waste in concrete manufacturing^39^, and the construction sector IS literature is still scarce. Addressing this limitation, the present study adopts five conceptual EIP scenarios specifically designed for the construction sector by Genc and Kurt^28^, in which construction serves as the ‘anchor industry’ (Robert U^40^ Ayres^1^. Potential symbiotic linkages for the sector are identified using data extracted from a database developed by the University of Cambridge’ Centre of Industrial Sustainability. This database was created under the EU-funded MAESTRI project, which aims to improve energy and resource management systems and enhance efficiency in process industries^41^. In the database, each symbiotic relationship is classified according to the industrial sectors of the participating firms, namely the waste generator and the waste receiver, using the European Commission’s NACE codes. Likewise, exchanged wastes and by-products are categorized through the European Waste Catalogue (EWC) codes. Therefore, identifying construction-related symbiosis requires considering not only sectors directly linked to construction, but also those that exchange waste materials with it. Based on this approach, a construction-oriented dataset is formed by evaluating all waste categories available within MAESTRI. The dataset encompasses the construction sector and its interconnections with industries that supply or receive waste streams. It comprises 48 sectors, 63 waste categories, and 170 potential symbiotic relationships.

**Fig. 1:**
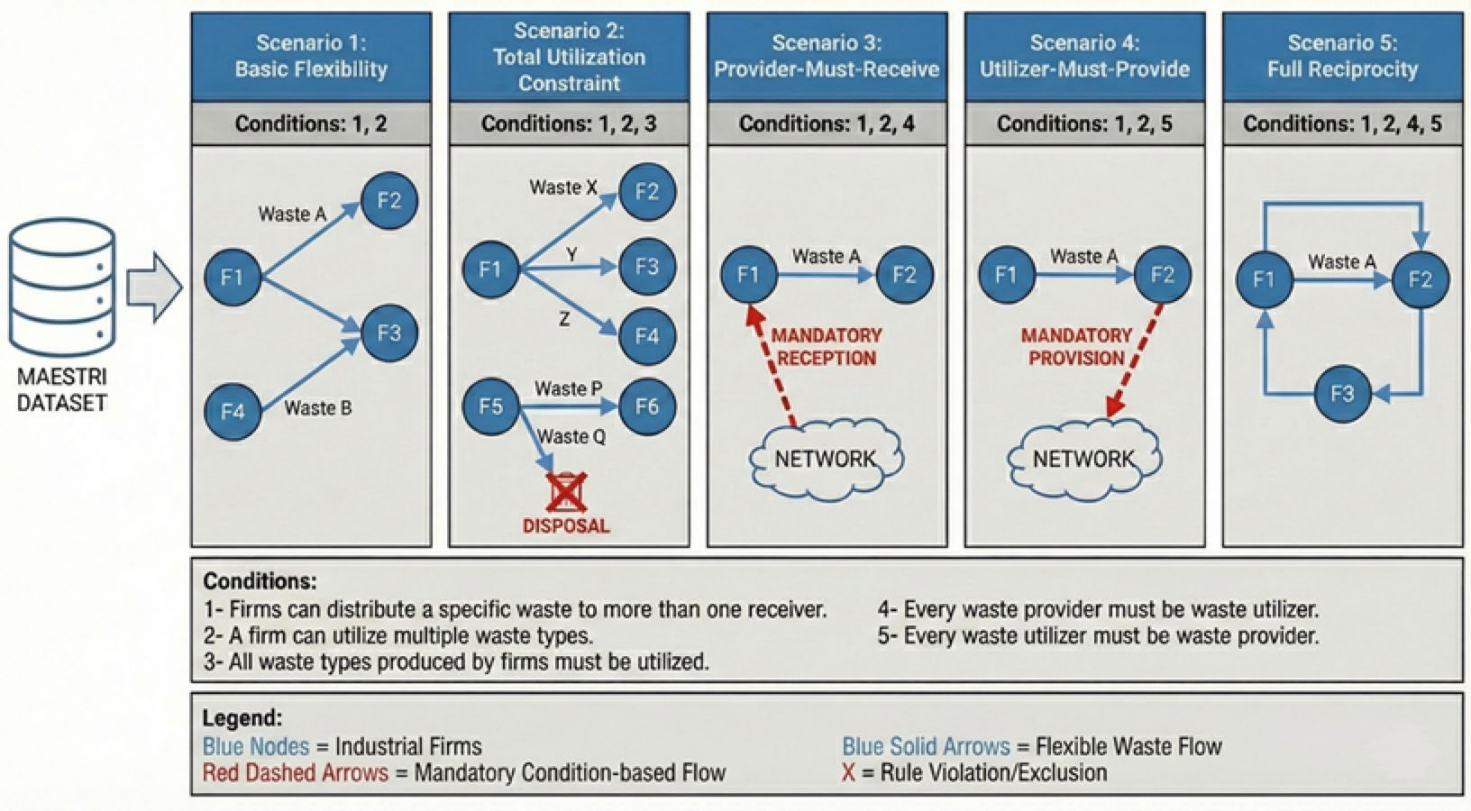
Schematic representation of scenario set-up and exchange logic.

The development of the five scenarios is grounded in key concepts of industrial ecology and industrial symbiosis, which highlight material and energy exchanges among firms as central mechanisms for achieving sustainability. Industrial ecology offers an overarching perspective for analysing the interactions and dependencies within industrial systems and supports the formation of networks that resemble natural ecosystems in terms of resource circulation and waste minimization (Ehrenfeld and Gertler^42^. In this context, the scenarios integrate a variety of symbiotic linkages in order to represent the different degrees of complexity and mutual dependence typically observed in effective EIPs. Prior research indicates that industrial networks that are both diverse and well-developed are generally more resilient and more efficient, as they can better respond to disruptions and changing conditions^43,44^. For this reason, the proposed scenarios deliberately differ in the intensity of interaction and collaboration between firms, thereby capturing a wide range of potential configurations. Additionally, the scenarios intentionally balance simplicity and complexity to enable evaluation under varying levels of industrial diversity and resource availability. While simpler structures with fewer exchanges provide a baseline for examining the core dynamics of symbiotic relationships, more complex designs involving multiple interconnected flows reflect the advanced integration typically found in mature EIPs^45^. This approach ensures that the study examines a broad spectrum of EIP designs and offers insights into both foundational and highly developed forms of industrial symbiosis. The detailed descriptions of the scenarios are presented in Table 1, while Fig. 1 provides a schematic illustration of the scenario set-up.

**Table 1:** Definition of industrial symbiosis scenarios and governing exchange rules.

Scenarios	Scenario description
Scenario 1	Firms generating waste that is suitable for symbiotic exchanges are allowed full flexibility in waste distribution. A given waste type can be divided and supplied to multiple partner firms in partial quantities. Likewise, firms may receive multiple waste types from other firms without being required to absorb the full quantity of each waste stream.
Scenario 2	Firms are allowed to distribute waste flexibly, meaning that the same waste type may be split among multiple partners and received in partial quantities. In addition, all waste types generated by a firm that are technically suitable for symbiotic use must be utilized within the network. If any eligible waste stream remains unused, the firm is excluded from participation in the eco-industrial park.
Scenario 3	Flexible waste distribution is permitted, allowing waste types to be split among multiple recipient firms and received in partial amounts. Furthermore, participation in waste exchange is reciprocal: any firm that supplies waste for symbiotic use must also receive waste inputs for its own production processes. The exchanged waste does not need to occur between the same pair of firms; incoming waste may originate from any partner within the network.
Scenario 4	Firms may distribute waste flexibly by splitting waste types among multiple recipients and receiving partial quantities from different partners. In this scenario, reciprocity is defined from the receiving side: any firm that receives waste for symbiotic purposes is also required to supply waste to the network. The outbound waste does not have to be sent back to the original supplier and may be directed to any firm within the eco-industrial park.
Scenario 5	Flexible waste distribution is allowed, enabling waste splitting and partial reception as in previous scenarios. In addition, full reciprocity is enforced: firms that supply waste must also receive waste, and firms that receive waste must also supply waste. These reciprocal exchanges are not required to be bilateral between the same firms; waste flows may occur among different partners within the network.

### Ecological network analysis (ENA)

**Fig. 2:**
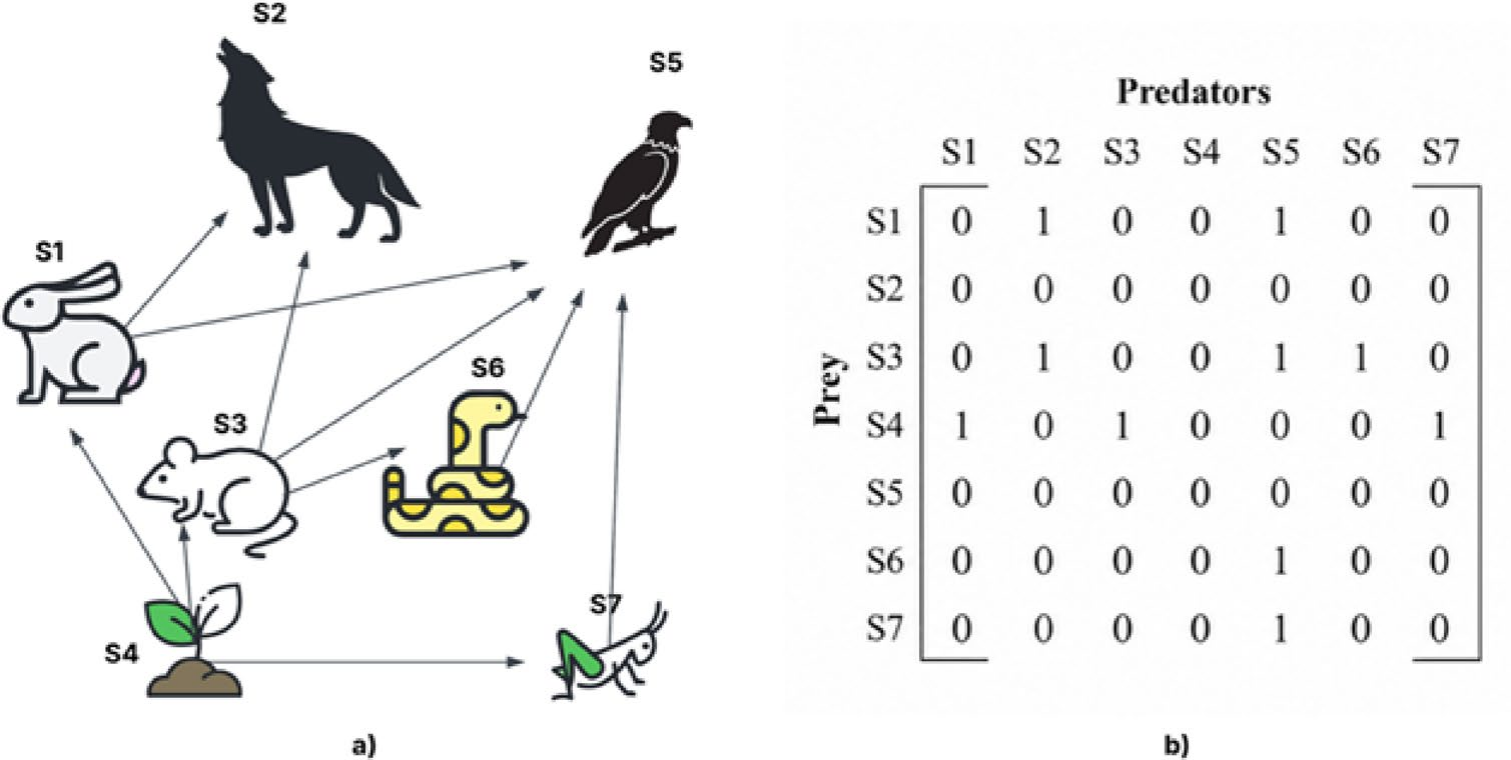
(**a**) The food web (FW) of a theoretical ecosystem, (**b**) The corresponding FW matrix [F]; where f_ij_ = 0 indicates no interaction between prey (*i*) and predator (*j*), and f_ij_ = 1 denotes a unidirectional interaction. The figure was manually created by the authors using Lucidchart, web-based version, Lucid Software Inc., accessed via https://lucid.app.

Energy and material exchanges in an ecological system can be modelled using a matrix referred to as the Food Web matrix [F]^46^, as illustrated in Fig. 2. Since each species in an ecosystem may act as a predator, a prey, or simultaneously both, the matrix [F] takes the form of a square matrix. To examine biological food webs, ecologists employ a set of structural metrics (Ulanowicz^47,48^. Designing alternative network configurations based on these metrics is consistent with conventional network design goals, including reducing emissions and costs while improving overall efficiency (A. C. Layton^49^, Mayer^50^, Reap^51^.

In this study, several structural food web (FW) metrics are adapted to characterize firm-level waste exchange networks across the analyzed scenarios using the Food Web matrix [F]. Species Richness (S_R_) represents the total number of species in a natural ecosystem and, in this context, corresponds to the total number of firms included in each scenario. Number of Links (N_L_) refers to the total number of direct interactions in the FW, represented by the nonzero entries in [F], and here it captures the total number of direct waste exchanges among firms by summing all nonzero elements of [F]. Link Density (L_D_), defined as the average number of flows (inputs and outputs) per species in ecological networks^52^, is used to indicate the ratio of waste exchanges to firms in the scenarios. Prey (N_prey_) denotes species that serve as food sources for others^53^; in the present industrial network context, however, the term is retained only as a food-web metric label and is interpreted as the source-side analogue of waste-providing firms, and calculated by summing nonzero row entries in [F], whereas Predator (N_predator_) refers to species that consume others^53^; in this study, it is interpreted as the receiving-side analogue of firms that receive waste or by-products for reuse, recovery, or processing, and computed by summing nonzero column entries in [F]. This terminology should therefore not be read as a literal predation analogy; industrial waste exchanges are closer to directed residual-resource exchanges and, in ecological terms, may resemble detrital or decomposer pathways where organisms feed on dead organic matter and waste^54^ more than classical predator–prey consumption. The Prey-to-Predator Ratio (P_r_) expresses the proportion of waste-producing firms to waste-receiving firms in each scenario. Generalization (G), defined as the average number of prey consumed per predator^55^, represents the average number of waste-producing firms connected to each waste-receiving firm, while Vulnerability (V), defined as the average number of predators per prey^55^, represents the average number of waste-receiving firms associated with each waste-producing firm. Because the present analysis uses a binary food-web matrix, G and V are calculated from the presence or absence of feasible exchange links only; they do not incorporate exchange quantities, biomass or material flows, interaction strengths, or link weights. Connectance (C) expresses the share of species pairs that are directly connected, reflecting the overall interaction intensity in ecological systems; in this study it denotes the ratio of realized direct waste exchanges to the total number of potentially feasible exchanges in the scenarios. Finally, Cyclicity (λ_max_) measures the degree of structural cycling in a FW(Fath and Halnes^56^ and is computed as the largest real eigenvalue of [F]. In this study, λ_max_ is used as a spectral topological indicator of potential closed-loop structure, rather than as a direct flow-based measure of cycling or material retention such as Finn’s Cycling Index^57^. Cyclicity (λ_max_) supports comparing natural and industrial systems^45^ and may take a value of 0 (no internal cycling), 1 (weak internal cycling), or greater than 1 (strong internal cycling)^58^. The formulations used to compute these metrics are presented in Supporting Information - S1.

### Mathematical modelling

The modelling approach adopted in this study is based on a limited biomimetic premise. Biological food webs are not treated here as direct evidence of sustainability that can be transferred wholesale to industrial systems. Rather, mature ecological networks are used as empirically observed structural reference systems that can inform the exploration of alternative industrial symbiosis configurations. Stability, resilience, and long-term sustainability in ecological systems depend not only on network topology, but also on interaction strengths, non-linear dynamics, environmental variability, and other process-level factors^29,31^. Therefore, the food-web metrics used in this study are interpreted as structural benchmarks, not as necessary or sufficient indicators of sustainability. Within this narrower interpretation, proximity to food-web benchmark values is used to evaluate whether construction-sector EIP scenarios can be configured to resemble selected ecological network properties. This assumption is consistent with biomimetic design work that uses ecological patterns as design analogies rather than direct performance guarantees^59,60^. Accordingly, the purpose of the model is to identify, within each of the five scenarios, the configuration whose metric values most closely approximate the selected biological food-web reference values and then compare these benchmark-proximate configurations with each other and with the selected biological reference set. The mean values derived from 55 ecological food webs reported by Layton et al.^45^ are used in this study as a detritus-inclusive biological food-web reference set for evaluating structural benchmark proximity^45^. This reference set is particularly relevant to the present study because the biological food webs compiled by the authors include documented detrital or recycling components, which are conceptually closer to industrial residual-resource exchanges than to a literal predator–prey analogy. Nevertheless, these average values should not be interpreted as a universal or ecosystem-independent food-web target. Food-web structure may vary with ecosystem type, spatial scale, trophic resolution, sampling intensity, and species richness. Accordingly, the benchmark values are used here as an aggregate structural reference drawn from an established biomimetic EIP comparison framework, rather than as a general ecological optimum. The average FW metric values calculated for these 55 food webs are provided in Table 2.

**Table 2:** The average FW metric values of 55 biological FWs^45^.

S_*R*_	*N*_L_	*N*_predator_	*N*_prey_	C	L_D_	*P*_*r*_	V	G	λ _max_
40	331	14	16	0,2	5,26	1,16	2,94	3,4	4,99

To reduce direct size dependence in comparisons across networks with different numbers of participating firms, metrics that are normalized by S_R_ and/or not directly dependent on network size (i.e., number of participating firms) are recommended for consideration^49,14^, namely L_D_, P_r_, V, G, and λ_max_. However, the use of these metrics does not imply that all size-related effects are fully eliminated, since the expected values and sensitivities of some network metrics may still vary with SR under different network-generation assumptions. Connectance captures the proportion of realized exchanges relative to all theoretically possible exchanges in a directed network and is therefore a compact descriptor of interaction “complexity” that directly shapes network topology and the distribution of interactions^61,26^. Although focusing on size-normalized metrics is essential for comparing networks of different sizes, connectance can still be consequential because it is structurally entangled with both SR and other ecological descriptors: for a given size, increases in $\:C\:$typically imply a denser interaction pattern (e.g., higher average links per node), which can systematically modify the conditions under which metrics derived from in- and out-degree patterns are realized (e.g., the balance between specialist and generalist interaction structures)^61,62^. Importantly, both unusually low and unusually high connectance can be undesirable when the goal is to emulate ecological benchmarks. Very low connectance indicates sparse networks with limited redundancy, which can amplify sensitivity to link or node removals, whereas higher connectance has been associated with greater robustness to secondary extinctions in empirical food-web analyses^26^. Conversely, classic complexity–stability theory highlights that increasing the density of interactions (i.e., increasing connectance in large systems) can push systems toward instability beyond critical thresholds, implying that excessively high connectance may also be counterproductive from an ecological-performance standpoint^29,31^. For these reasons, connectance is not only a descriptive indicator but a potential driver of trade-offs among ecological benchmark targets; accordingly, to explicitly test its influence on the optimization outcomes, we divided the optimization experiments into two parallel sets: (i) formulations in which connectance is included alongside the size-normalized ecological benchmarks, and (ii) formulations in which connectance is omitted, retaining only the other normalized benchmarks. This two-track design is implemented consistently for each of the five construction industry symbiosis scenarios, yielding paired “C-included” and “C-excluded” optimization models per scenario, and is intended to examine the conditioning role of connectance in optimized network morphology rather than to claim full control over all SR-related effects.

In natural ecosystems, cannibalism refers to situations in which an organism consumes all or part of another individual of the same species. One reason why ecological food webs typically exhibit stronger interconnectedness and higher internal cycling (i.e., closed-loop structures) than EIPs is the occurrence of cannibalism (Layton et al., 2016), which functions as a self-loop within the network^63^. In an industrial context, processes that reintroduce self-generated waste streams as inputs can be conceptually compared to this biological mechanism, since a firm effectively “consumes” its own by-products (Layton et al., 2016). Such self-loop exchanges are known to contribute positively to cyclicity in industrial networks^17^. Therefore, self-loop relationships are explicitly considered in this study, and the values of C are calculated using Formula 2 (See Supporting Information-S1) in comparisons.

A mixed-integer non-linear programming model (MINLP) is proposed. In this model, 4 different objective function types are considered independently. Hereafter, they are referred to as OFT1, OFT2, OFT3, and OFT4. The objective functions are formulated to minimize the discrepancy between the optimized network’s metric values and the selected biological reference values. In other words, the model selects the exchange configuration whose food-web metric values are closest to the corresponding reference values, thereby producing a network configuration with the lowest structural deviation under the selected objective-function formulation. Due to non-linearity and size of problem, the model cannot be solved with current solver and computer technology. Therefore, a genetic algorithm (GA) is proposed to obtain effective feasible solutions within small computational times. The MINLP model is presented to represent the problem mathematically.

The notations used in mathematical model and solution methodologies are presented below:

**Parameters:**

- *\:N* Set of industrial firms.
- *\:K* Set of waste types.
- *\:M* Number of performance metrics.
- *\:i,j* index for industrial firms $\:i,j\in\:N$.
- *\:k* index for waste types $\:k\in\:K$.
- *\:m* index for performance metrics $\:m\in\:M$.
- $\:{e}_{ik}$ binary parameter equal to 1 if waste *k* can be received by firm *i*, and 0 otherwise.
- $\:{c}_{ik}$ binary parameter equal to 1 if waste *k* can be produced by firm *i*, and 0 otherwise.
- $\:{z}_{m}^{I}$ Ideal value of performance metric *m*.

**Decision variables:**

- $\:{S}_{ijk}$ binary variable that takes the value 1 if waste *k* can be transferred from firm *i* to firm *j*, and 0 otherwise.
- $\:{R}_{ij}$ binary variable that takes the value 1 if there exists a connection from firm *i* to firm *j*, and 0 otherwise.
- $\:{Y}_{i}$ binary variable that takes the value 1 if firm *i* is constructed in the eco-park, and 0 otherwise.
- $\:{G}_{i}$ binary variable that takes the value 1 if firm *i* is a waste giver, and 0 otherwise.
- $\:{T}_{i}$ binary variable that takes the value 1 if firm *i* is a waste taker, and 0 otherwise.
- $\:{\lambda\:}_{max}$ Maximum eigen value.
- $\:{V}_{j}$ Elements of corresponding eigenvector of $\:{{\uplambda\:}}_{\mathrm{m}\mathrm{a}\mathrm{x}}$.
- $\:{z}_{m}$ Obtained value of performance metric *m*.

*Methods for the convergence to ideal values*:

1. Minimize the sum of absolute deviations relative to the ideal values for each performance metric.

$$\:minimize\:\sum\:_{m\in\:M\:}\frac{\left|{z}_{m}-{z}_{m}^{I}\right|}{{z}_{m}^{I}}$$

(2) Minimize the sum of absolute deviation between obtained and ideal performance metric.

$$\:minimize\:\sum\:_{m\in\:M\:}\left|{z}_{m}-{z}_{m}^{I}\right|$$

(3) Minimize the Euclidean distance between obtained and ideal performance metrics.

$$\:minimize\:\sqrt{\sum\:_{m\in\:M\:}{\left({z}_{m}-{z}_{m}^{I}\right)}^{2}}$$

(4) Minimize the sum of ratios considering the maximum and minimum value obtained for performance metrics.

$$\:minimize\:\sum\:_{m\in\:M\:}\frac{\left|{z}_{m}-{z}_{m}^{I}\right|}{\underset{m\in\:M}{\mathrm{max}}{z}_{m}-\underset{m\in\:M}{\mathrm{min}}{z}_{m}}$$

$\:\underset{m\in\:M}{\mathrm{max}}{z}_{m}$ and $\:\underset{m\in\:M}{\mathrm{min}}{z}_{m}$ values are obtained with following methods:

- Each metric independently aimed to reach its ideal value with solution methodology, and then $\:\lVertM\lVert$ solutions are obtained ($\:\lVertM\lVert$ is the total number of performance metrics).
- Each metric value is calculated according to these solutions. Then, maximum and minimum values of them are reported as $\:\underset{m\in\:M}{\mathrm{max}}{z}_{m}$ and $\:\underset{m\in\:M}{\mathrm{min}}{z}_{m}$ values.

The four objective function types should be interpreted as alternative aggregation schemes for measuring structural deviation from the selected biological reference values, rather than as claims of ecological equivalence among the metrics. The metrics considered in this study differ in their ecological meaning, numerical scale, variability, and sensitivity to network changes. Therefore, an equal numerical deviation in one metric does not necessarily imply the same ecological consequence as an equal deviation in another metric. In this study, equal weighting is used as a transparent baseline modelling choice that enables comparison across scenarios and objective-function formulations without introducing additional subjective weighting assumptions. OFT1 partially addresses numerical scaling by using relative deviations, and OFT4 introduces a range-based normalization; however, neither approach should be interpreted as resolving differences in ecological importance among metrics.

*The constraint set of the MINLP is given below*:

Constraint (1) defines the first performance metric which is Cyclicity.

1 $$\:{z}_{1}={{\uplambda\:}}_{\mathrm{m}\mathrm{a}\mathrm{x}}$$

Constraint (2) calculates the performance metric Connectance (C).

2 $$\:{z}_{2}=\frac{{\sum\:}_{i\in\:N}{\sum\:}_{j\in\:N}{R}_{ij}}{{\left(\sum\:_{i\in\:N}{Y}_{i}\right)}^{2}}\:\:\:\:\:\:\:\:\:\:\:\:$$

Constraint (3) is used to calculate Link density.

3 $$\:{z}_{3}=\frac{{\sum\:}_{i\in\:N}{\sum\:}_{j\in\:N}{R}_{ij}}{\sum\:_{i\in\:N}{Y}_{i}}\:$$

Prey to Predator Ratio is calculated by Constraint (4).

4 $$\:{z}_{4}=\frac{{\sum\:}_{i\in\:N}{G}_{i}}{\sum\:_{i\in\:N}{T}_{i}}$$

Constraint (5) calculates the Vulnerability.

5 $$\:{z}_{5}=\frac{{\sum\:}_{i\in\:N}{\sum\:}_{j\in\:N}{R}_{ij}}{\sum\:_{i\in\:N}{G}_{i}}$$

Constraint (6) defines the performance metrics Generalization.

6 $$\:{z}_{6}=\frac{{\sum\:}_{i\in\:N}{\sum\:}_{j\in\:N}{R}_{ij}}{\sum\:_{i\in\:N}{T}_{i}}\:$$

Constraint (7) calculates the maximum eigen value (λ_max_). The left of Constraint 7 represents the obtained connection matrix. Both side with $\:{Y}_{i}$ to satisfy the constraint when the firm is not opened in the EIP. Both side of equation will be zero if the firm *i* is not opened.

7 $$\:\sum\:_{i\in\:N}{{\mathrm{Y}}_{\mathrm{i}}{\mathrm{Y}}_{\mathrm{j}}\mathrm{R}}_{\mathrm{i}\mathrm{j}}{\mathrm{V}}_{\mathrm{j}}={{\mathrm{Y}}_{\mathrm{i}}\mathrm{V}}_{\mathrm{i}}{{\uplambda\:}}_{\mathrm{m}\mathrm{a}\mathrm{x}},\:\:\:i\in\:N\:\:$$

Constraint set (8) ensures that waste *k* cannot be transferred from firm *i* to firm *j* if firm *i* is not opened or it cannot produce waste *k*.

8 $$\:{S}_{ijk}\le\:{c}_{ik}{Y}_{i}\:\mathrm{f}\mathrm{o}\mathrm{r}\:i,j\in\:N;k\in\:K\:$$

Constraint set (9) guarantees that waste *k* cannot be transferred from firm *i* to firm *j* if firm *j* is not opened or it cannot receive waste *k*.

9 $$\:{S}_{ijk}\le\:{e}_{jk}{Y}_{j}\:\mathrm{f}\mathrm{o}\mathrm{r}\:i,j\in\:N;k\in\:K\:$$

Constraint set (10) guarantees that waste *k* must be transferred from firm *i* to firm *j* if firm *i* and *j* are opened, and *i* can produce and *j* can receive waste *k*.

10 $$\:{S}_{ijk}\ge\:{c}_{ik}{Y}_{i}+{e}_{jk}{Y}_{j}-1\:\mathrm{f}\mathrm{o}\mathrm{r}\:i,j\in\:N;k\in\:K$$

Constraint set (11) states that waste *k* can be transferred from firm *i* to firm *j* if firm *i* and *j* are opened, and *i* can produce and *j* can receive waste *k*.

11 $$\:2{S}_{ijk}\le\:{c}_{ik}{Y}_{i}+{e}_{jk}{Y}_{j}\:\mathrm{f}\mathrm{o}\mathrm{r}\:i,j\in\:N;k\in\:K$$

Constraint set (12) ensures that no connection exists from firm *i* to firm *j* if any waste is not transferred.

12 $$\:{R}_{ij}\le\:\sum\:_{k\in\:K}{S}_{ijk}\:\mathrm{f}\mathrm{o}\mathrm{r}\:i,j\in\:N$$

Constraint set (13) states that firm *i* can be opened if it has a connection.

13 $$\:\sum\:_{j\in\:N}\left({R}_{ij}+{R}_{ji}\right)\ge\:{Y}_{i}\:\mathrm{f}\mathrm{o}\mathrm{r}\:i\in\:N$$

Constraint set (14) ensures that firm *i* can be waste provider if there exists at least one connection from firm *i* to other firms.

14 $$\:\sum\:_{j\in\:N}{R}_{ij}\ge\:{G}_{i}\:\mathrm{f}\mathrm{o}\mathrm{r}\:i\in\:N$$

Constraint set (15) guarantees that firm *i* can be waste receiver if there exists at least one connection to firm *i* from other firms.

15 $$\:\sum\:_{j\in\:N}{R}_{ji}\ge\:{T}_{i}\:\mathrm{f}\mathrm{o}\mathrm{r}\:i\in\:N$$

Constraint set (16) guarantees that a waste of the opened firm must be received by any firm.

16 $$\:{c}_{ik}{Y}_{i}\le\:\sum\:_{j\in\:N}{e}_{jk}{Y}_{j}\:\mathrm{f}\mathrm{o}\mathrm{r}\:i\in\:N;k\in\:K$$

Constraint set (17) guarantees that a firm must receive waste if it transfers a type of waste to any firm.

17 $$\:\sum\:_{j\in\:N}{R}_{ij}\le\:\left|N\right|\sum\:_{j\in\:N}{R}_{ji}\:\mathrm{f}\mathrm{o}\mathrm{r}\:i\in\:N\:$$

Constraint set (18) ensures that a firm must transfer waste to any firm if it receives a type of waste from any firm.

18 $$\:\sum\:_{j\in\:N}{R}_{ji}\le\:\left|N\right|\sum\:_{j\in\:N}{R}_{ij}\:\mathrm{f}\mathrm{o}\mathrm{r}\:i\in\:N$$

Constraint set (19) represents the binary restrictions of the decision variables.

$$\:Y_{i} ,R_{{ij}} ,S_{{ijk,}} T_{i} ,G_{i} \in \:\left\{ {{\mathrm{0,1}}} \right\}\,for\,\:i,j \in \:N;k \in \:K$$

However, it is crucial to note that the constraint sets utilized in these scenarios vary and are enumerated in Table 3.

**Table 3:** Constraint set used in scenarios.

Scenario	1	2	3	4	5
Used constraint sets	7, 8, 9, 10, 12, 13, 14, 15, 19	7, 8, 9, 10, 12, 13, 14, 15, 16, 19	7, 8, 9, 10, 12, 13, 14, 15, 17, 19	7, 8, 9, 10, 12, 13, 14, 15, 18, 19	7, 8, 9, 10, 12, 13, 14, 15, 17, 18, 19

### Genetic algorithm (GA)

**Fig. 3:**
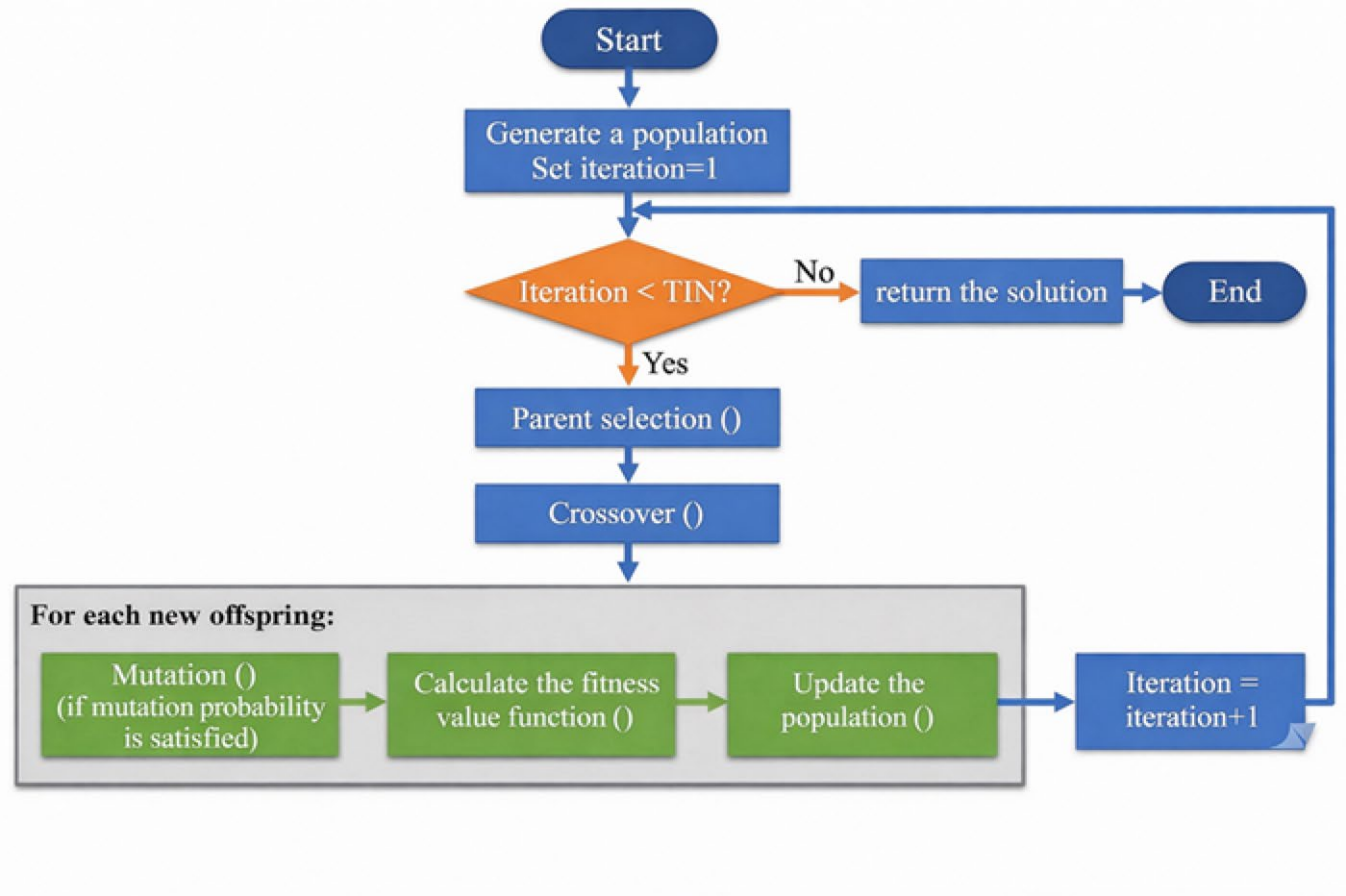
The flow of GA.

In the Genetic Algorithm (GA), only the existence information of firms is stored in memory. Moreover, all possible connections between firms must be established when the firms exist in the industrial park, due to constraint sets (10) and (11). The proposed GA is a classical meta-heuristic search method developed by Holland^64^. The structure of the proposed GA is illustrated in Fig. 3, and the details of the operators used in the GA are presented in the following subsections.

#### Chromosome representation

Chromosomes in the GA are binary-based and represent the construction decisions of firms in the industrial zone. Each gene in a chromosome indicates the existence of a firm and can take only binary values. A value of one means that the firm is constructed, whereas zero indicates non-construction. Consequently, the length of each chromosome equals the number of alternative firms for the industrial zone.

#### Decoding and fitness value function

After obtaining a chromosome, all possible connections among the opened firms are established. Based on these connections and the collected firm information, the performance metrics can be calculated. The selected objective function value is used as the fitness value of the chromosome; therefore, a smaller fitness value indicates a better solution. If any of the specified conditions are violated, the fitness value of the chromosome is assigned a sufficiently large number. In OFT 4, the metric with same minimum and maximum values is not considered due to having zero at the denominator.

#### Finding the initial population

The population size (PS) is predetermined and specified before the execution of the algorithm. The initial population is generated randomly. First, a threshold value is randomly selected, and firms are opened based on this value. For each firm, a random number is generated, and the firm is opened (the gene takes the value of one) if the generated number is greater than the threshold. This process is repeated until the number of feasible chromosomes reaches the population size.

#### Parent selection

Two chromosomes are selected from the population to generate new offspring using the crossover operator. Tournament selection is employed for parent selection. For each parent, two random individuals are chosen, and the one with the better fitness value is selected for reproduction.

#### Crossover

The crossover operation is applied to generate new offspring from two parent chromosomes. In this GA, a uniform crossover operator is used for reproduction. Uniform crossover employs a threshold crossover probability to decide which parent’s gene is inherited by the offspring. This threshold probability is randomly determined at each crossover by generating a random number between 0 and 1. Then, for each gene of the offspring, another random number between 0 and 1 is generated. If this value is less than the threshold crossover probability, the gene of the first offspring is taken from the first parent; otherwise, it is taken from the second parent. The second offspring is generated using the same procedure.

#### Mutation

To enhance chromosome diversity, a mutation operator is applied. For each newly generated chromosome, a random mutation probability between 0.001 and 0.1 is assigned, and the mutation operator is executed based on this probability. During mutation, one gene is selected at random and its value is flipped, changing from 0 to 1 or from 1 to 0.

#### Update population

Whenever a new chromosome is generated, the population is updated to ensure improvement. The algorithm employs a full elitism strategy, in which only the best chromosomes are retained and the worst one is removed. If a newly created offspring is worse than all existing members, it is directly discarded.

#### Termination procedure

The algorithm’s process for generating new populations is limited by a predetermined total iteration number (TIN), which also serves as the termination criterion for the GA. The search procedure stops when this iteration limit is reached, and the resulting solution is then reported.

#### Parameter tuning for genetic algorithm

The proposed algorithm largely relies on randomness, and most of its parameters are determined randomly. However, two parameters—the population size (PS) and the total iteration number (TIN)—must be specified before executing the algorithm. To identify suitable parameter values, three levels are tested for each parameter. The average objective function value is used as the evaluation criterion. Since the algorithm has a short computation time, solution time is not considered. The tested parameter levels and their average performances are presented in Table 4.

**Table 4:** The parameter levels and their performance in the genetic algorithm.

		*PS*		
		10	30	50
TIN	5000	2.3763	1.9775	1.9347
	10,000	2.3635	1.9923	**1.9336**
	15,000	2.2652	2.0388	1.9807

According to the results, the algorithm achieves its best performance when the TIN is set to 10,000 and the PS is set to 50. Therefore, the results presented in this study are based on these parameter settings.

## Results

This section reports the optimized eco-industrial park (EIP) network configurations obtained under five scenario formulations and four optimization function types (OFTs), evaluated both with and without the explicit inclusion of connectance (*C*) in the optimization model.

**Table 5:** Structural properties and derived food web metrics for all optimization function types (OFTs), scenarios, and C-inclusion settings.

Obj. Func. Type	C-included	Scenario	Obj. Func. Value	S_*R*_	*N*_L_	*N*_prey_	*N*_predator_	C	L_D_	*P*_*r*_	V	G	λ_max_
1	No	1	0.709	40	85	29	25	0.053	2.125	1.160	2.931	3.400	4.442
		**2**	**0.682**	**39**	**85**	**29**	**25**	**0.056**	**2.179**	**1.160**	**2.931**	**3.400**	**4.527**
		3	0.837	7	24	7	7	0.490	3.429	1.000	3.429	3.429	4.108
		4	0.844	7	21	7	6	0.429	3.000	1.167	3.000	3.500	3.201
		5	0.783	11	37	11	11	0.306	3.364	1.000	3.364	3.364	4.342
	Yes	**1**	**0.694**	**14**	**38**	**13**	**11**	**0.194**	**2.714**	**1.182**	**2.923**	**3.455**	**4.295**
		2	1.296	30	73	24	21	0.081	2.433	1.143	3.042	3.476	4.527
		3	1.061	16	55	14	16	0.215	3.438	0.875	3.929	3.438	4.752
		4	0.810	14	40	14	12	0.204	2.857	1.167	2.857	3.333	3.594
		5	1.920	8	25	8	8	0.391	3.125	1.000	3.125	3.125	3.594
2	No	1	3.257	26	62	21	18	0.092	2.385	1.167	2.952	3.444	4.672
		2	3.472	32	75	25	22	0.073	2.344	1.136	3.000	3.409	4.527
		3	3.164	11	37	11	11	0.306	3.364	1.000	3.364	3.364	4.342
		**4**	**3.092**	**12**	**38**	**12**	**11**	**0.264**	**3.167**	**1.091**	**3.167**	**3.455**	**4.342**
		5	3.121	12	44	12	12	0.306	3.667	1.000	3.667	3.667	4.615
	Yes	1	3.411	28	65	22	19	0.083	2.321	1.158	2.955	3.421	4.672
		2	3.644	36	86	28	24	0.066	2.389	1.167	3.071	3.583	4.672
		3	3.457	9	32	9	9	0.395	3.556	1.000	3.556	3.556	4.363
		**4**	**3.155**	**12**	**38**	**12**	**11**	**0.264**	**3.167**	**1.091**	**3.167**	**3.455**	**4.342**
		5	3.261	12	42	12	12	0.292	3.500	1.000	3.500	3.500	4.401
3	No	1	1.945	17	63	16	16	0.218	3.706	1.000	3.938	3.938	4.752
		2	2.434	27	84	21	23	0.115	3.111	0.913	4.000	3.652	4.752
		**3**	**1.785**	**10**	**39**	**10**	**10**	**0.390**	**3.900**	**1.000**	**3.900**	**3.900**	**4.615**
		**4**	**1.785**	**10**	**39**	**10**	**10**	**0.390**	**3.900**	**1.000**	**3.900**	**3.900**	**4.615**
		**5**	**1.785**	**10**	**39**	**10**	**10**	**0.390**	**3.900**	**1.000**	**3.900**	**3.900**	**4.615**
	Yes	1	1.928	15	58	14	15	0.258	3.867	0.933	4.143	3.867	4.752
		2	2.435	27	84	21	23	0.115	3.111	0.913	4.000	3.652	4.752
		**3**	**1.795**	**10**	**39**	**10**	**10**	**0.390**	**3.900**	**1.000**	**3.900**	**3.900**	**4.615**
		**4**	**1.795**	**10**	**39**	**10**	**10**	**0.390**	**3.900**	**1.000**	**3.900**	**3.900**	**4.615**
		5	1.808	11	43	11	11	0.355	3.909	1.000	3.909	3.909	4.546
4	No	1	1.655	38	86	29	25	0.060	2.263	1.160	2.966	3.440	4.615
		2	2.858	37	87	28	25	0.064	2.351	1.120	3.107	3.480	4.672
		3	1.261	12	44	12	12	0.306	3.667	1.000	3.667	3.667	4.615
		4	1.618	9	27	9	8	0.333	3.000	1.125	3.000	3.375	3.524
		**5**	**0.839**	**12**	**44**	**12**	**12**	**0.306**	**3.667**	**1.000**	**3.667**	**3.667**	**4.615**
	Yes	1	1.328	39	87	29	25	0.057	2.231	1.160	3.000	3.480	4.527
		2	2.109	30	73	24	21	0.081	2.433	1.143	3.042	3.476	4.527
		3	1.470	12	42	12	12	0.292	3.500	1.000	3.500	3.500	4.401
		4	1.065	14	43	14	12	0.219	3.071	1.167	3.071	3.583	4.106
		**5**	**0.969**	**12**	**42**	**12**	**12**	**0.292**	**3.500**	**1.000**	**3.500**	**3.500**	**4.401**

Significant values are in bold.

Table 5 synthesizes the structural properties and food-web–inspired network metrics obtained for each combination of optimization function type (OFT), scenario, and connectance (C) setting (C-excluded vs. C-included). Bold values in the objective-function column indicate the best-performing solution within each OFT and C-inclusion setting. Within each OFT and C-setting, the minimum objective-function value identifies the best-performing solution. It should be noted that the reported best-performing configurations represent the minimum scalar objective value under each OFT and scenario setting. They should therefore be interpreted as representative benchmark-proximate solutions under the selected aggregation rule, rather than as the only practically relevant design alternatives. In addition, objective-function values should not be compared directly across OFTs, because each OFT uses a different deviation aggregation and scaling structure. In particular, OFT2 sums absolute deviations from the biological reference values without benchmark-based or range-based normalization, which explains why its objective-function values are numerically larger than others. To provide additional information on near-optimal alternatives and convergence behavior, the top five final-generation GA solutions for each scenario, objective function type, and C-inclusion setting are reported in a Table, while the evolution of objective-function values across iterations is shown in a Figure both can be found in Supporting Information – S3. The corresponding scenario selections and resulting network sizes used in this study are as follows:

*OFT 1*

- C-excluded: best solution occurs under Scenario 2 (objective = 0.682), yielding a comparatively large network (*S*_*R*_ = 39, *N*_*L*_ = 85).
- C-included: best solution occurs under Scenario 1 (objective = 0.694), producing a mid-sized network (*S*_*R*_ = 14, *N*_*L*_ = 38).

*OFT 2*

- C-excluded: best solution occurs under Scenario 4 (objective = 3.092), with *S*_*R*_ = 12 and *N*_*L*_ = 38.
- C-included: best solution occurs under Scenario 4 (objective = 3.155), with *S*_*R*_ = 12 and *N*_*L*_ = 38.

*OFT 3*

- C-excluded: best solutions are obtained under Scenario 3, Scenario 4 and Scenario 5 (objective = 1.785), all producing a compact network (*S*_*R*_ = 10, *N*_*L*_ = 39).
- C-included: best solution occurs under Scenario 3 and Scenario 4 (objective = 1.795), with *S*_*R*_ = 10 and *N*_*L*_ = 39.

*OFT 4*

- C-excluded: best solution occurs under Scenario 5 (objective = 0.839), yielding *S*_*R*_ = 12 and *N*_*L*_ = 44.
- C-included: best solution occurs under Scenario 5 (objective = 0.969), yielding *S*_*R*_ = 12 and *N*_*L*_ = 42.

Across the four OFTs, the selected best-performing scenarios vary by objective-function type and C-inclusion setting: Scenario 4 is selected under some C-included cases (OFT 2 and 3), whereas the best-performing C-excluded solutions vary more strongly across scenarios (Scenario 2 for OFT 1; Scenario 4 for OFT 2; Scenario 3/4/5 for OFT 3; Scenario 5 for OFT 4). These selections indicate that no single scenario or OFT provides uniform superiority across all food-web metrics; instead, performance is expressed through measurable trade-offs among link density, the balance between waste-providing and waste-receiving firms, connectance, generalization, vulnerability, and cyclicity.

The best-performing solutions produce distinct structural and metric patterns. Link density (LD) in the best-performing solutions spans from relatively low values in OFT 1 to markedly higher values under OFT 3 and also OFT 4. For example, the best OFT 3 solutions yield L_D_ = 3.90 (C-excluded: Scenarios 3, 4, and 5; C-included: Scenarios 3 and4), whereas best solutions under OFT 1 / C-excluded and OFT 2 / C-excluded yield L_D_ = 2.18 and 3.17, respectively. Prey-to-predator ratio (P_r_) in best-performing solutions clusters in a narrow range for most OFTs, with multiple best solutions taking P_r_ = 1.00 (OFT 3 best cases; OFT 4 best cases), while other best cases remain close (P_r_ = 1.16 for OFT 1/C-excluded/Scenario 2; P_r_ = 1.18 for OFT 1/C-included/Scenario 1; P_r_ = 1.09 for OFT 2 best cases). Vulnerability (V) and generalization (G) display the strongest contrast between OFT 3 and the other OFTs in the best-performing cases. The best OFT 3 solutions report V = 3.90 and G = 3.90, while best-performing solutions in other OFTs are lower (V = 2.93, G = 3.40 for OFT 1/C-excluded/Scenario 2; V = 3.16, G = 3.45 for OFT 2 best cases; and V = 3.67, G = 3.67 for OFT 4/C-excluded/Scenario 5). Cyclicity (λ_max_) varies across OFTs and C-settings, with best-performing solutions ranging from 4.29 (OFT 1 / C-included / Scenario 1) up to 4.61 (OFT 3 best cases; also, OFT 4/C-excluded/Scenario 5), while the best OFT 4 / C-included solution yields λ_max_ = 4.40. Across all selected best-performing configurations, L_D_ and λ_max_ remain below the selected biological reference values, indicating that the optimization does not achieve biological benchmark levels for network density or structural cycling under the current construction-sector feasibility constraints. Connectance (C), when reported as an outcome (including C-excluded cases where it is not optimized), spans a broad range across the best-performing solutions: for instance, C = 0.056 in the best OFT 1 / C-excluded solution (Scenario 2), but C = 0.39 in the best OFT 3 solutions (C-excluded: Scenarios 3, 4, and 5; C-included: Scenarios 3–4). Under C-included best solutions, C ranges from 0.19 (OFT 1/Scenario 1) to 0.39 (OFT 3/Scenario 3–4), with OFT 2/Scenario 4 and OFT 4/Scenario 5 yielding intermediate values (0.26 and 0.29, respectively).

**Fig. 4:**
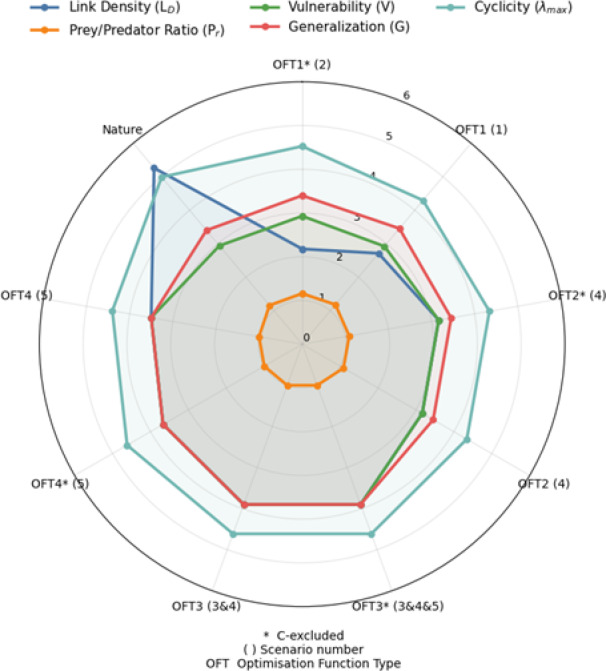
Food-web metric values for the selected best-performing configurations across optimization function types compared with mean reference values from 55 biological food webs.

**Fig. 5:**
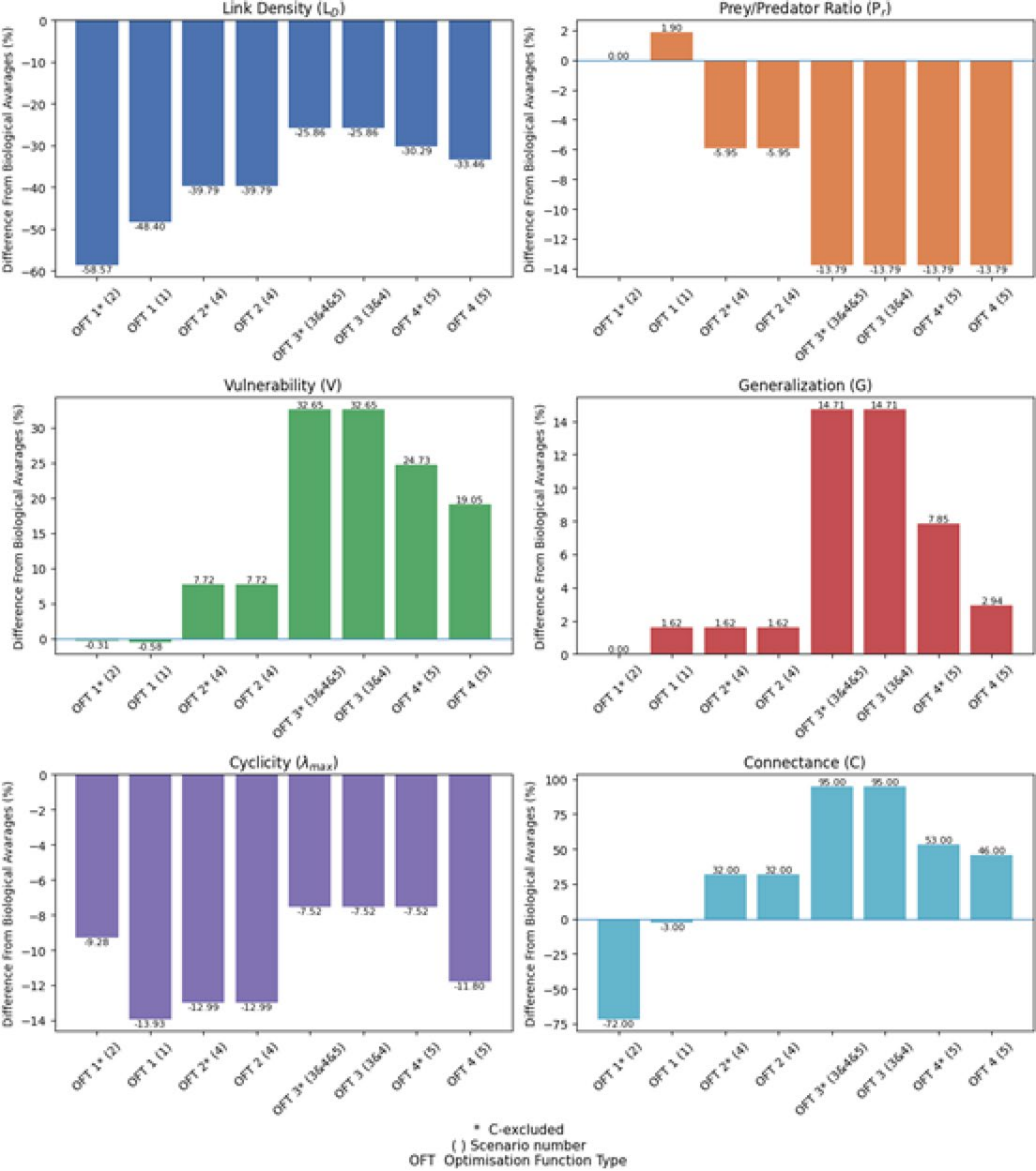
Percentage differences of food web metrics relative to biological reference values for the selected best-performing configurations under each optimization function type (OFT).

To synthesize the multi-metric outcomes, Fig. 4 plots the selected food-web metric values for the best-performing solutions across OFTs and scenarios, alongside the corresponding biological food-web reference values. The configurations shown in Fig. 4 were selected from Table 5 by choosing, for each OFT and C-inclusion setting, the scenario with the lowest objective-function value. Figure 5 reports the corresponding percentage differences from biological reference values for each metric, calculated as the deviation from each biological reference value divided by that reference value, enabling a metric-by-metric inspection. Negative percentage differences indicate that the optimized EIP configuration remains below the biological reference value for the corresponding metric, whereas positive values indicate that the metric exceeds the reference value. This interpretation helps distinguish metrics that approach the selected biological reference values from those that remain persistently below or above them across the selected configurations. Link density (L_D_) is lower than the biological benchmark for all compared best-performing solutions, with negative deviations ranging from approximately − 25.86% to − 58.57% depending on OFT and scenario. The smallest magnitude deviation occurs for the OFT 3 cases (both C-excluded and C-included selections), which show the least negative departure (− 25.86%), whereas the largest negative deviation is observed for OFT 1, C-excluded, Scenario 2 (− 58.57%). Prey-to-predator ratio P_r_ shows a clearer separation between OFTs: values are strongly negative for most configurations, spanning approximately − 5.95% to − 13.79%, with the OFT 3 and OFT 4 selections reaching the largest negative deviations (− 13.79%). In contrast, OFT 1, C-included, Scenario 1 shows a positive offset (+ 1.90%), while OFT 1, C-excluded, Scenario 2 is at 0.00%. Vulnerability (V) exhibits modest negative deviations for OFT 1 selections (− 0.31% to − 0.58%), moderate positive deviations for OFT 2 selections (+ 7.72%), and substantially larger positive deviations for OFT 3 selections (+ 32.65% for both C settings). OFT 4 selections also remain strongly positive, though lower than OFT 3 (+ 24.73% for OFT 4, C-excluded, Scenario 5 and + 19.05% for OFT 4, C-included, Scenario 5). Generalization (G) follows a similar pattern: values are near zero to small positive in OFT 1 (0.00% to + 1.62%) and OFT 2 (+ 1.62%), but increase strongly for OFT 3 (+ 14.71%) and remain elevated for OFT 4 (+ 7.85% and + 2.94%). Cyclicity (λ_max_) is consistently below the biological benchmark across all displayed solutions, with deviations spanning approximately − 7.52% to − 13.93%. The least negative values occur for the OFT 3 selections and OFT 4, C-excluded, Scenario 5 (− 7.52%), while the most negative deviation is observed for OFT 1, C-included, Scenario 1 (− 13.93%). Connectance (C) shows the widest spread of deviations, ranging from a large negative difference (− 72.00% for OFT 1, C-excluded, Scenario 2) to very large positive differences (+ 95.00% for both OFT 3 selections). OFT 2 selections are moderately positive (+ 32.00%), while OFT 4 selections remain strongly positive (+ 53.00% and + 46.00%), and OFT 1, C-included, Scenario 1 is only slightly negative (− 3.00%). Synthesizing these results, Figs. 3 and 4 indicate that best-performing solutions produce distinct metric signatures by OFT and C-setting, with the strongest contrasts observed in V, G, and C (particularly the marked elevation for OFT 3 and OFT 4). At the same time, several metrics (notably L_D_ and λ_max_) remain consistently below the biological benchmark across all selections, indicating that closeness to biological averages varies by metric rather than improving uniformly across all metrics simultaneously.

**Fig. 6:**
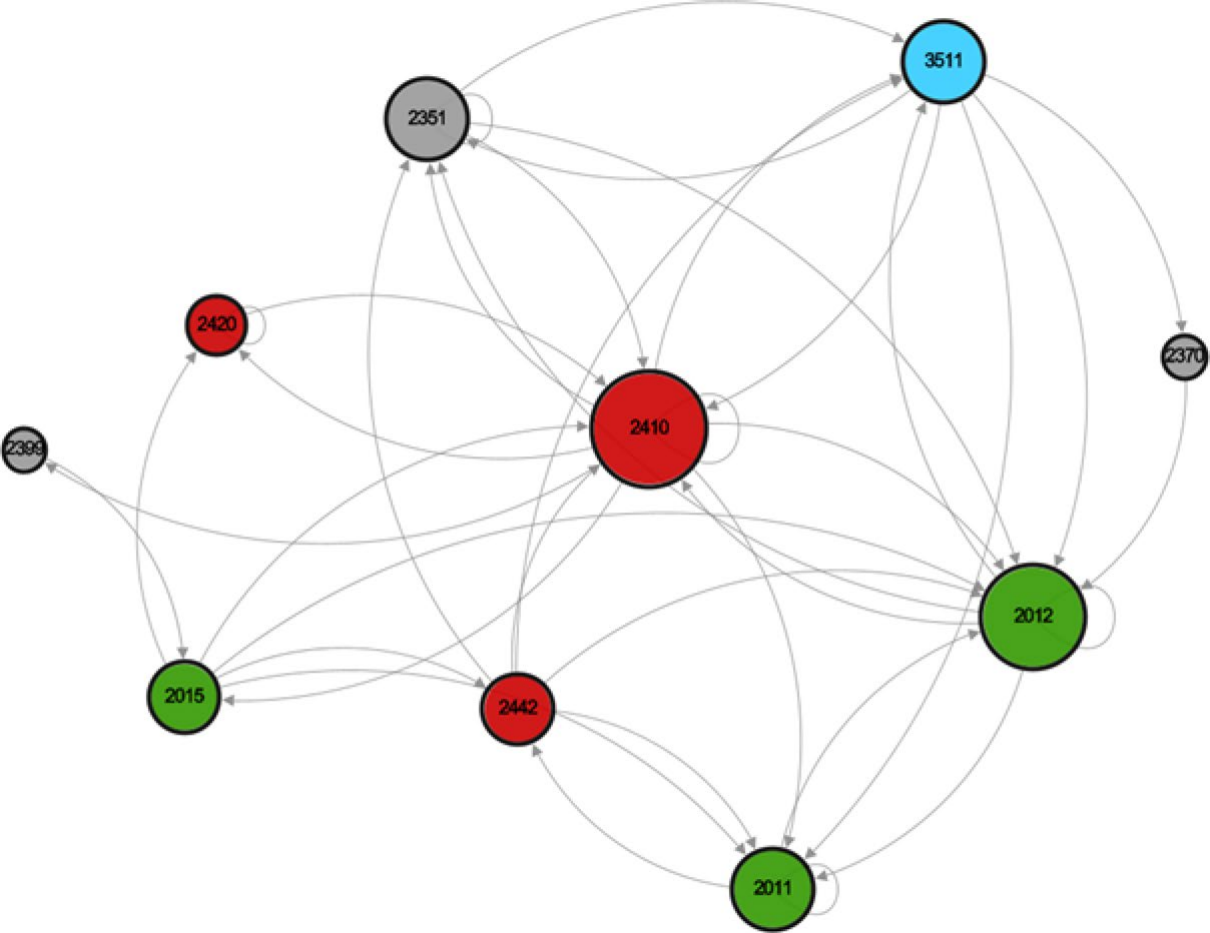
Directed symbiotic exchange network of the selected EIP configuration (OFT 3, C-included, Scenario 4).

The industrial symbiosis network obtained under OFT 3 with connectance included (Scenario 4) is presented for illustration purposes. The NACE codes and corresponding NACE branches of the waste-supplying and waste-receiving firms in this EIP, together with the number of symbiotic exchange relationships among them, are reported in Table 6. A graphical representation of the symbiotic network structure of this EIP is shown in Fig. 6. Equivalent detailed representations for all other best-performing solutions are provided in Supporting Information-S2.

Table 6; Fig. 6 together reveal a highly structured and unevenly distributed pattern of symbiotic exchanges within the selected EIP configuration. The network is clearly organized around a small number of highly connected industrial sectors that function as structural hubs. The manufacture of basic iron and steel and ferro-alloys form the structural core of the network, maintaining numerous outgoing and incoming exchanges with cement production, electricity generation, chemical manufacturing, and metal processing activities. The manufacture of dyes and pigments appears as a key waste-receiving sector, absorbing diverse by-products from chemicals, metals, and energy-related processes, while the electricity sector plays a bridging role by both utilizing industrial residues and linking otherwise separate material streams. In contrast, activities such as stone cutting and finishing and other non-metallic mineral product manufacturing occupy more peripheral positions, participating in fewer and more specialized exchanges. The presence of several intra-sector exchanges further indicates internal reuse alongside inter-firm symbiosis. Overall, the network exhibits a clear core–periphery structure typical of industrial symbiosis systems, where energy-intensive and material-processing industries anchor circulation, and more specialized sectors connect to this core through a limited number of symbiotic pathways.

**Table 6:** Sectoral composition and number of symbiotic exchange relationships among firms in the selected EIP configuration (OFT 3, C-included, Scenario 4).

Waste producer (Prey)		Waste receiver (Predator)		Number of symbiotic relationship
NACE code	NACE branches	NACE code	NACE branches	
2011	Manufacture of industrial gases	2442	Aluminium production	1
2011	Manufacture of industrial gases	2011	Manufacture of industrial gases	1
2011	Manufacture of industrial gases	2012	Manufacture of dyes and pigments	1
2012	Manufacture of dyes and pigments	3511	Production of electricity	4
2012	Manufacture of dyes and pigments	2410	Manufacture of basic iron and steel and of ferro-alloys	2
2012	Manufacture of dyes and pigments	2351	Manufacture of cement	1
2012	Manufacture of dyes and pigments	2012	Manufacture of dyes and pigments	2
2012	Manufacture of dyes and pigments	2011	Manufacture of industrial gases	3
2015	Manufacture of fertilisers and nitrogen compounds	2442	Aluminium production	2
2015	Manufacture of fertilisers and nitrogen compounds	2420	Manufacture of tubes, pipes, hollow profiles and related fittings, of steel	1
2015	Manufacture of fertilisers and nitrogen compounds	2410	Manufacture of basic iron and steel and of ferro-alloys	1
2015	Manufacture of fertilisers and nitrogen compounds	2012	Manufacture of dyes and pigments	1
2015	Manufacture of fertilisers and nitrogen compounds	2011	Manufacture of industrial gases	1
2351	Manufacture of cement	2351	Manufacture of cement	1
2351	Manufacture of cement	2410	Manufacture of basic iron and steel and of ferro-alloys	1
2351	Manufacture of cement	2012	Manufacture of dyes and pigments	1
2351	Manufacture of cement	3511	Production of electricity	1
2370	Cutting, shaping and finishing of stone	2012	Manufacture of dyes and pigments	1
2399	Manufacture of other non-metallic mineral products	2015	Manufacture of fertilisers and nitrogen compounds	1
2410	Manufacture of basic iron and steel and of ferro-alloys	2015	Manufacture of fertilisers and nitrogen compounds	1
2410	Manufacture of basic iron and steel and of ferro-alloys	2351	Manufacture of cement	3
2410	Manufacture of basic iron and steel and of ferro-alloys	2399	Manufacture of other non-metallic mineral products	1
2410	Manufacture of basic iron and steel and of ferro-alloys	2410	Manufacture of basic iron and steel and of ferro-alloys	4
2410	Manufacture of basic iron and steel and of ferro-alloys	3511	Production of electricity	3
2410	Manufacture of basic iron and steel and of ferro-alloys	2420	Manufacture of tubes, pipes, hollow profiles and related fittings, of steel	2
2410	Manufacture of basic iron and steel and of ferro-alloys	2012	Manufacture of dyes and pigments	1
2410	Manufacture of basic iron and steel and of ferro-alloys	2011	Manufacture of industrial gases	1
2420	Manufacture of tubes, pipes, hollow profiles and related fittings, of steel	2420	Manufacture of tubes, pipes, hollow profiles and related fittings, of steel	2
2420	Manufacture of tubes, pipes, hollow profiles and related fittings, of steel	2410	Manufacture of basic iron and steel and of ferro-alloys	2
2442	Aluminium production	2410	Manufacture of basic iron and steel and of ferro-alloys	3
2442	Aluminium production	2351	Manufacture of cement	5
2442	Aluminium production	2012	Manufacture of dyes and pigments	1
2442	Aluminium production	2011	Manufacture of industrial gases	1
2442	Aluminium production	3511	Production of electricity	1
3511	Production of electricity	2370	Cutting, shaping and finishing of stone	1
3511	Production of electricity	2351	Manufacture of cement	2
3511	Production of electricity	2410	Manufacture of basic iron and steel and of ferro-alloys	2
3511	Production of electricity	2012	Manufacture of dyes and pigments	3
3511	Production of electricity	2011	Manufacture of industrial gases	2

## Discussion

This study set out to examine how selected food-web structural targets can steer the optimization of construction-sector eco-industrial park (EIP) networks, and crucially how alternative objective-function formulations (OFTs), scenario rules, and the explicit inclusion of connectance metric jointly shape the emergent network structure. The comparative evidence reported in Table 5 and visualized in Figs. 4 and 5 indicates that structural benchmark proximity is not a single or uniform outcome but a configuration-dependent property. The same scenario can move some metrics toward biological averages while simultaneously displacing others, and the same OFT can behave differently when connectance is treated as a target versus left to emerge endogenously. These findings are consistent with the broader industrial symbiosis (IS) and industrial ecology (IE) literature, which repeatedly emphasizes that network performance depends on both the availability of exchanges and the rules that govern how exchanges are formed (Chertow^7,35^; Ehrenfeld and Gertler^65,34,66^), as well as with ecological network theory showing that food-web structure is jointly constrained by size, connectance, and organizational principles rather than by any single metric in isolation^26,67,55^; Ulanowicz^68,48,69^.

A central pattern in Fig. 5 is the asymmetry in how readily different metrics approach biological averages. Across the compared best solutions, link density (L_D_) remains substantially below biological averages in every case, while prey-to-predator ratio (P_r_) shows comparatively smaller departures in some configurations but is not uniformly close to the benchmark across the full set, several OFT–scenario pairs exhibit pronounced negative deviations. The same figure shows that cyclicity (λ_max_) is consistently below biological averages, whereas vulnerability (V) and generalization (G) can be either close to biological averages or substantially higher depending on the OFT–scenario pair. This uneven convergence difficulty aligns with the ecological observation that different structural properties scale differently with network size and sampling, and that connectance-linked properties often shift as systems become more (or less) densely wired^26,67,10,55,69^. In an EIP context, it also echoes recurring practical constraints. Many early-stage designs can create plausible exchange matches, but not necessarily the breadth of realized links that would raise L_D_ toward ecological baselines without violating feasibility and supply-demand compatibility^35,34,66^.

The clearest methodological implication is that OFTs are not interchangeable even when they nominally pursue the same “closeness-to-ideal” aim. The metric-value comparison in Fig. 4 indicates that each OFT systematically prioritizes certain dimensions of network structure. When combined with the metric-by-metric deviations in Fig. 5, two tendencies stand out: (1) OFTs that reduce deviations in a largely additive manner can keep several metrics near target while leaving LD persistently low, and (2) the Euclidean-distance style aggregation (OFT 3) can produce overshoot in some metrics, especially when connectance is not directly constrained. For example, the solutions associated with OFT 1 and OFT 2 keep P_r_ and some robustness-related indicators relatively near the biological averages, yet L_D_ remains far below. This suggests that the optimization landscape allows cheap improvements in ratio-type indicators without requiring the large increase in realized links that would lift L_D_. In both ecological food webs and industrial symbiosis networks, ratio-type metrics can be adjusted by reorganizing roles (predator/prey analogues, or senders/receivers) without necessarily creating many new links^26,15^; Ulanowicz^68^). The most striking deviations in Fig. 5 occur under the OFT 3 cases. V and G rise well above biological averages, and connectance becomes dramatically larger than the benchmark for the OFT 3 solutions in general, rather than only in C-excluded configurations. Such overshoot is consistent with a known feature of squared-distance aggregation. It penalizes large deviations strongly, encouraging the solver to spend structural changes where they most efficiently shrink the largest squared gaps, which can unintentionally inflate other metrics. In network design terms, adding or rewiring links can rapidly change role distributions (generalists vs. specialists) and exposure patterns (vulnerability), particularly in directed networks where in- and out-neighborhood sizes change nonlinearly with added links^26,10^. In IS design, similar trade-offs have been observed when optimization emphasizes a narrow subset of targets or aggregates heterogeneous goals into a single scalar measure: improving one dimension (e.g., utilization or exchange coverage) can reshape the network topology and shift other emergent properties^23,24,66^. Taken together, these findings reinforce that the OFT is not merely a computational choice but a *structural bias* that channels the solver toward particular network morphologies. In particular, the OFT 3 pattern in Figs. 4 and 5 shows how aggressively minimizing a global distance measure can drive multiple structure-related metrics (V, G, and C) far above ecological reference levels, even while other metrics such as L_D_ and λ_max_ remain below the benchmark. This is aligned with broader calls in the EIP optimization literature to scrutinize objective aggregation carefully, particularly when the desired end state is defined by multiple structural and functional criteria^23,24^. From a decision-support perspective, these results also indicate that no single configuration should be interpreted as universally preferable across all planning contexts. A stakeholder prioritizing cyclicity may select a different configuration than one prioritizing connectance control, lower network density, or broader firm participation. Therefore, the scalar OFT results are most useful as structured comparisons of how alternative aggregation rules shape network outcomes, while practical EIP planning would benefit from presenting a set of non-dominated alternatives.

A major contribution of this study is that C is not just another metric; it is a control-like property that can indirectly shift many other metrics because it constrains how many realized interactions can exist relative to the possible interaction set. In ecological network theory, connectance and size are foundational drivers of food-web structure, influencing path redundancy, potential cycling, and the distribution of specialist/generalist roles^26,55,48^. Accordingly, treating connectance as endogenous (C-excluded) versus targeted (C-included) creates a meaningful experimental split, and the results support this design choice empirically. Figure 5 shows that several “C-included” solutions keep connectance close to biological reference values, whereas the most extreme overshoots in connectance occur under OFT 3 configurations in general rather than being limited to a single C-excluded case. This indicates that, without an explicit connectance target, the optimization can gravitate toward overly connected solutions, especially under OFT structures that reward rapid reduction in composite distance. From an industrial symbiosis standpoint, such high-connectance designs may be questionable in practice because real EIP exchanges face transaction costs, quality compatibility requirements, coordination burdens, and governance constraints that limit the feasible number of connections per firm (Chertow^7,35,33,34^). The results therefore support a key planning implication; including connectance is not only ecologically motivated but also acts as a proxy for real-world limits on exchange proliferation, preventing the optimizer from achieving structural benchmark proximity by unrealistically densifying the network.

Although all five scenarios share the baseline flexibility assumption (splitting waste types and allowing partial fulfillment), the additional rules impose different participation symmetries and closure constraints that plausibly reshape topology. Consistent with the IS literature, requirements that firms both send and receive exchanges can increase reciprocity and reduce purely one-directional participation, thereby potentially pushing the network toward more integrated structures under specific OFT and C-inclusion settings^35^; Ehrenfeld and Gertler^65,33^. This matches the ecological analogy where trophic networks are structured by mutual constraints on feeding roles and the availability of resource and consumer links^55^; Ulanowicz^68^). The scenario set can therefore be interpreted as progressively tightening network closure conditions. Scenario 1 allows participation without additional closure constraints, likely permitting more peripheral or one-sided firms. Scenario 2 introduces a strong closure condition (all usable waste must be used), effectively disallowing partial or selective participation; this should prune the feasible firm set and can reduce network size while increasing coherence among remaining links, an outcome compatible with the observed sensitivity of size- and connectance-related metrics. Scenarios 3 and 4 impose *directional reciprocity*; senders must receive (Scenario 3) or receivers must send (Scenario 4). In directed-network terms, these constraints reduce degree asymmetries and can systematically change the in-degree/out-degree distributions, which in turn affects vulnerability and generalization patterns^26,10^. Scenario 5 combines both reciprocity constraints, pushing the system toward more strongly interdependent structures. In ecological network analysis, alterations in degree distributions and the balance of specialist/generalist roles are known to influence vulnerability and generalization (via the distribution of consumers per resource and resources per consumer) and also to shape the propensity for cyclic pathways (Fath and Halnes^27^, Ulanowicz^68^). These mechanism-based expectations are consistent with the fact that the best-performing solutions plotted in Figs. 4 and 5 span multiple scenarios rather than collapsing to a single “best” scenario across all metrics. This provides a coherent interpretation of why the best scenario for one metric can differ from the best for another: a scenario that increases role symmetry may improve P_r_ or stabilize G yet simultaneously move L_D_ and λ_max_ in an unfavorable direction if feasible links remain limited by the underlying exchange-compatibility matrix. Such trade-offs are well-known in EIP design, where feasibility and acceptability conditions can dominate the space of possible exchanges even when sustainability objectives encourage broader connectivity^23,24,34,66^.

### Implications for construction-sector EIPs and bio-inspired benchmarking

The construction sector is frequently characterized by heterogeneous material streams, variable quality and contamination constraints, and fragmented supply chains, all of which complicate sustained, high-frequency exchange patterns^70^. Against this backdrop, the persistent LD deficit relative to biological benchmarks can be read as a realistic structural signal, even under flexible splitting rules, achieving link densities comparable to the selected biological reference values may be difficult without either expanding the candidate firm set, increasing the diversity of usable waste categories, or introducing enabling infrastructure and governance mechanisms that lower transaction barriers, consistent with EIP success-factor studies and IS facilitation research^33,34^. This interpretation is reinforced by Fig. 5, where all best-performing solutions remain well below the biological benchmark for LD, regardless of OFT or scenario. Meanwhile, the sensitivity of connectance to OFT choice and C-inclusion underscores that bio-inspired is not a single recipe. Using ecological benchmarks as targets requires explicit decisions about which ecological properties are treated as constraints versus emergent outcomes^26,48^ a point that is increasingly emphasized in biomimicry-guided sustainable design frameworks^51,20^. Finally, because industrial ecology has long framed industrial systems through metabolism metaphors and ecosystem analogies^1,71,72^, the present results help operationalize that analogy in structural and metric-specific terms: they demonstrate empirically that movement toward selected food-web structural reference values depends on the mathematical representation of closeness (OFT), the institutional logic of participation (scenario constraints), and the explicit governance of density (including connectance). This triangulation of metrics, scenarios, and objective structures offer a practical roadmap for how construction-focused EIP planning can use ecological benchmarks without inadvertently creating topologies that are either too sparse to be effective or too dense to be feasible in practice^23,35,26,24^.

### Limitations

Several limitations should be considered when interpreting the findings. First, the networks are derived from a scenario- and database-constrained feasibility space; therefore, achievable structure is bounded by the available firm–waste compatibility and the scenario rules. More specifically, the optimization is based on a fixed waste–sector compatibility matrix derived from the available construction-sector symbiosis data. As a result, the findings should not be interpreted as universally generalizable to all construction-sector EIPs or to other regional and industrial contexts without further validation using additional datasets. Second, for the biomimetic interpretation of the results, this study does not assume that natural ecosystems are inherently sustainable or that structural similarity to a food web is sufficient to produce sustainability, resilience, or stability in an industrial network. The metrics considered here capture only selected topological properties; they do not represent interaction strengths, flow magnitudes, exchange reliability, non-linear feedback, adaptation, environmental variability, or institutional dynamics, all of which may strongly condition system stability and persistence^29,31^. Therefore, the reported benchmark proximity should be interpreted as structural benchmark alignment rather than as evidence of superior environmental performance, economic viability, governance feasibility, or practical implementability. Third, the results may be sensitive to the selected biological reference set and to the normalization and weighting assumptions embedded in the OFTs. Although the use of equal weights provides a transparent baseline for comparing scenario and OFT effects, it does not imply that deviations in connectance, link density, vulnerability, generalization, prey–predator ratio, and cyclicity have equivalent ecological meanings or consequences. Alternative weighting schemes based on expert judgement, empirical metric variability, or ecosystem-specific reference ranges may lead to different optimization outcomes. Fourth, the outcomes are also conditional on the scenario rules and on whether connectance is treated as an explicit objective component or left to emerge from the optimized network structure. Therefore, the reported configurations should be read as scenario- and formulation-specific structural outcomes, not as universally optimal EIP designs. Finally, the current model uses binary network structure only; it does not incorporate material flow quantities, economic costs, waste-quality compatibility, technological readiness, contractual feasibility, or operational uncertainty. Including these factors may substantially alter the feasible exchange set and the resulting optimal configurations.

## Conclusion and future research

This study shows that optimizing construction-sector eco-industrial park networks toward selected ecological structural benchmarks is feasible, but the outcome is strongly contingent on (1) how closeness is mathematically defined and, (2) how participation is governed through scenario rules. Across the tested configurations, the best solutions consistently demonstrate that structural benchmark proximity is not achieved by improving all indicators simultaneously; rather, it emerges from negotiated trade-offs among density, role balance, specialization patterns, and cycling potential. In particular, solutions selected under different optimization function types (OFTs) reached comparable overall proximity in some metrics while diverging sharply in others, confirming that the OFT itself functions as a design lever that steers the optimizer toward distinct network morphologies. A second key finding is the structural importance of explicitly including connectance as a target. When connectance was excluded, the optimizer could achieve apparent improvements in several metrics by drifting toward extreme connectivity regimes (either sparse or overly dense subnetworks), depending on the OFT. When connectance was included, solutions more consistently converged toward the selected connectivity reference levels and produced more balanced metric profiles. This pattern highlights that connectance acts as a structuring constraint that limits extreme connectivity regimes and conditions the co-variation of other metrics, rather than serving as direct evidence of ecological realism or sustainability. At the scenario level, reciprocity-based participation rules influenced which configurations could approach the selected benchmark structure under specific OFT and connectance-inclusion settings. However, the updated results indicate that no single scenario uniformly dominates across all metrics; instead, different scenarios support different aspects of benchmark proximity depending on the OFT and the inclusion of connectance. By contrast, scenarios that allowed more one-sided participation or imposed strict “all eligible waste must be used” requirements tended to push the optimizer toward solutions that improved some dimensions while worsening others, reflecting the sensitivity of network structure to closure constraints and firm participation symmetry. Metric-wise, the results indicate that link density and cyclicity do not reach the selected biological food-web reference values under the current feasibility constraints, even in the best-performing solutions. Meanwhile, the balance between waste-providing and waste-receiving firms tended to remain comparatively close to benchmarks across many selected cases. Together, these outcomes suggest that while role balance can be aligned through feasible rewiring under scenario constraints, increasing the breadth of realized exchanges and strengthening cyclic structure remain the most challenging aspects of structurally informed bio-inspired EIP design for the construction dataset considered.

This study advances biomimetic EIP research by (1) operationalizing structural benchmark proximity as a multi-metric network optimization problem under explicit scenario logic, (2) demonstrating that objective-function choice systematically shapes the resulting topology and metric trade-offs, and (3) empirically clarifying the structural role of connectance as a conditioning constraint rather than a purely descriptive statistic. The paired comparison of connectance-included versus connectance-excluded formulations provides a transparent framework for diagnosing when good solutions are driven by closer agreement with selected food-web structural reference values versus by structural shortcuts. For planners and decision-makers in construction-sector symbiosis initiatives, the results offer two early-stage structural design implications. First, aiming for food-web-informed structural targets requires more than maximizing the number of exchanges. Scenario rules that promote reciprocal participation may contribute to more balanced and benchmark-consistent designs under certain objective-function and connectance-inclusion settings, although no single scenario uniformly optimizes all metrics simultaneously. Second, including an explicit connectivity control can prevent designs that are theoretically close in a subset of metrics but implausible to implement due to excessive or insufficient linkage density. In this sense, the proposed framework supports early-stage screening of scenario policies and optimization settings before moving to detailed engineering design.

Future work should prioritize methods that make multi-metric trade-offs transparent and decision-relevant (e.g., Pareto-based reporting or multi-criteria selection), while enriching the current structural framework with flow magnitudes, quality constraints, and economic/environmental performance indicators to better connect benchmark proximity to real-world feasibility and benefits. Future research should also examine alternative objective-function structures that better reflect differences in ecological meaning, variability, and sensitivity among food-web metrics. This may include expert-informed metric weights, variance- or range-based weighting schemes, ecosystem-specific benchmark ranges, and multi-objective formulations that report Pareto-efficient solution sets rather than collapsing all deviations into a single scalar objective value. Such extensions would allow decision-makers to examine trade-offs among ecological network metrics more explicitly and to select configurations according to context-specific planning priorities. In addition, robustness under uncertainty and system dynamics should be evaluated, and the approach should be tested across other construction datasets and regional contexts, alongside implementation evidence, to assess transferability and inform policy and practice mechanisms that enable additional link formation and cycling without undermining feasibility.

## Supplementary Information

Below is the link to the electronic supplementary material.

Supplementary Material 1

Supplementary Material 2

Supplementary Material 3

## Data Availability

Data are available from the corresponding author upon reasonable request.

## Declarations

**The of using Generative AI and AI-assisted technologies**.

During the preparation of this work, the authors used Generative AI tools in order to synthesis of information from diverse sources, paraphrasing and language editing. After using these tools, the authors reviewed and edited the content as needed and take full responsibility for the content of the publication.
