# Supplementary material for: Biologically inspired optimization of construction sector eco industrial park networks using food web metrics
Source: Sci Rep. 2026 May 24;16:23693. doi: 10.1038/s41598-026-54667-x (PMC13424639; doi:10.1038/s41598-026-54667-x)
Supplement: Supplementary file 1 — Supplementary Material 1 [file 41598_2026_54667_MOESM1_ESM.docx]

| **FW Metric** | **In a Natural Ecosystem** | **In This Study** | **Calculation Formula** |
| --- | --- | --- | --- |
| Species Richness (S_R_) | Total number of species (Gotelli and Colwell, 2001). | Total number of firms in an EIP scenario. | - |
| Number of Links (N_L_) | Total number of direct relations between species in a food web [F]. | Total number of direct waste flows between firms within an EIP scenario. | $N_{L}=\sum_{i=1}^{m} \sum_{j=1}^{n} f_{\mathrm{ij}}$ |
| Link Density (L_D_) | Average number of flows into or out of each species (Ulanowicz et al., 2014). | Average number of waste flow per firm within an EIP scenario. | $L_{D}=\frac{N_{L}}{S_{R}}$ |
| Prey (N_prey_) | A species consumed by other species (Hardy and Graedel, 2002). | Waste-providing firms in an EIP scenario. | $f_{r}\left( i \right)=\left\{ \begin{aligned} 0 for \sum_{j=1}^{n} f_{\mathrm{ij}}=0 \\ 1 for \sum_{j=1}^{n} f_{\mathrm{ij}}>0 \end{aligned} \right.$  $N_{\mathrm{prey}}=\sum_{i=1}^{m} f_{r}\left( i \right)$ |
| Predator (N_predator_) | A species that consumes other species (Hardy and Graedel, 2002). | Waste-receiving firms in an EIP scenario. | $f_{c}\left( j \right)=\left\{ \begin{aligned} 0 for \sum_{i=1}^{m} f_{\mathrm{ij}}=0 \\ 1 for \sum_{i=1}^{m} f_{\mathrm{ij}}>0 \end{aligned} \right.$  $N_{\mathrm{predator}}=\sum_{j=1}^{n} f_{c}\left( j \right)$ |
| Prey-to-Predator Ratio (P_r_) | The ratio of the number of prey to the number of predators. | The ratio of the number of waste-providing firms to waste-receiving firms within an EIP scenario. | $P_{r}=\frac{N_{\mathrm{prey}}}{N_{\mathrm{predator}}}$ |
| Generalization (G) | The average number of prey consumed per predator (Reap, 2009). | The number of waste-providing firms interacted per waste-receiving firm within an EIP scenario. | $G=\frac{N_{L}}{N_{\mathrm{predator}}}$ |
| Vulnerability (V) | The average number of predators per prey (Reap, 2009). | The number of waste-receiving firms per waste-providing firm within an EIP scenario. | $V=\frac{N_{L}}{N_{\mathrm{prey}}}$ |
| Connectance (C) | Fraction of pairs of species that directly interact (Yodzis, 1980). | The number of realized direct waste exchanges within an EIP scenario divided by the total number of theoretically possible directed exchanges. | $\left( 1 \right) C=\frac{N_{L}}{S_{R}\left( S_{R}-1 \right)}$  $\left( 2 \right) C=\frac{N_{L}}{S_{R}^{2}}$ |
| Cyclicity (λ_max_) | A spectral topological indicator of structural cycling potential in a food web (Fath and Halnes, 2007). | A spectral topological indicator of potential closed-loop structure in an EIP scenario | $\det\left( F- \lambda I \right)= 0 \to$λ_max_ |

**Table.** Food Web (FW) Metrics Employed for This Study

**References**

Fath, B.D., Halnes, G., 2007. Cyclic energy pathways in ecological food webs. Ecol Modell 208, 17–24. https://doi.org/10.1016/J.ECOLMODEL.2007.04.020

Gotelli, N.J., Colwell, R.K., 2001. Quantifying biodiversity: procedures and pitfalls in the measurement and comparison of species richness. Ecology Letters 4, 379–391. https://doi.org/10.1046/j.1461-0248.2001.00230.x

Hardy, C., Graedel, T.E., 2002. Industrial Ecosystems as Food Webs. J Ind Ecol 6, 29–38. https://doi.org/10.1162/108819802320971623

Reap, J.J., 2009. Holistic biomimicry:  a biologically inspired approach to environmentally benign engineering.

Ulanowicz, R.E., Holt, R.D., Barfield, M., 2014. Limits on ecosystem trophic complexity: insights from ecological network analysis. Ecol Lett 17, 127–136. https://doi.org/10.1111/ele.12216

Yodzis, P., 1980. The connectance of real ecosystems. Nature 284, 544–545. https://doi.org/10.1038/284544a0
